# A new species of *Tangius* from north India (Coleoptera, Staphylinidae, Pselaphinae)

**DOI:** 10.3897/zookeys.346.6233

**Published:** 2013-11-01

**Authors:** Zi-Wei Yin, Li-Zhen Li

**Affiliations:** 1 Department of Biology, College of Life and Environmental Sciences, Shanghai Normal University, 100 Guilin Road, Shanghai, 200234, P. R. China; 2 Department of Biology, School of Life Sciences, East China Normal University, 3663 North Zhongshan Road, Shanghai,200062, P. R. China

**Keywords:** Batrisina, taxonomy, *Tangius*, new species

## Abstract

*Tangius indicus* **sp. n.** (Batrisitae: Batrisini) is described and illustrated from the Indian States of Meghalaya (Khasi Hills, type locality) and West Bengal (Darjeeling). Specimens of the new species are similar to those of the recently described *T. glabellus* Yin & Li from Tibet, Southwest China, and can be separated only by minor differences of the male features.

## Introduction

The batrisine genus *Tangius* Yin & Li (Yin et al. 2012) was recently established for a single species *Tangius glabellus* Yin & Li, from southeast Tibet (= Xizang A. R.). Characters distinguishing it from the allied genera *Dendrolasiophilus* Nomura, *Maajappia* Nomura, and *Songius* Yin & Li were described.

When visiting the Natural History Museum of Geneva, Switzerland (May, 2013), the first author sorted a small series of north Indian *Tangius* specimens out of the large pselaphine collection. A second species of the genus, closely allied to *Tangius glabellus*, was recognized based on differences in male characters. In this paper we describe the new species, provide illustrations of its major diagnostic features, and distinguish it from the previously described *Tangius glabellus*.

## Material and methods

The type series is housed in the Muséum d’histoire naturelle de la Ville de Genève, Switzerland (**MHNG** – G. Cuccodoro).

The collection data of the referred material are quoted verbatim. A slash (/) is used to separate different labels. Authors’ notes are included in ‘[]’.

Measurements are in millimeters. The following abbreviations are applied: AL–length of the abdomen along the midline; AW–maximum width of the abdomen; EL–length of the elytra along the sutural line; EW–maximum width of the elytra; HL–length of the head from the anterior clypeal margin to the occipital constriction; HW–width of the head across eyes; PL–length of the pronotum along the midline; PW–maximum width of the pronotum. Length of the body equals HL + PL + EL + AL + length of the occipital constriction.

## Taxonomy

### Key to males of *Tangius*

**Table:** 

1	Antennomeres VII–IX subcylindrical, as long as wide to slightly transverse (Fig. 2A); mesotibiae distinctly concave along mesal margin before preapical denticle (Fig. 2F); apex of median lobe broadly rounded on the left side (Fig. 2M). (north India: Meghalaya, West Bengal)	*Tangius indicus* sp. n.
–	Antennomeres VII–IX asymmetrically trapezoidal, distinctly transverse (Yin et al. 2012: 59, fig. 6); mesotibiae have straight mesal margins before preapical denticle (Yin et al. 2012: 59, fig. 13; Fig. 2G); apex of median lobe angularly rounded on the left side (Fig. 2L). (Southwest China: Xizang)	*Tangius glabellus* Yin & Li

### Tangius indicus

Yin & Li sp. n.

http://zoobank.org/49049A5A-07D6-452C-A3AA-FF5E0689E84B

http://species-id.net/wiki/Tangius_indicus

**Figure 1.**
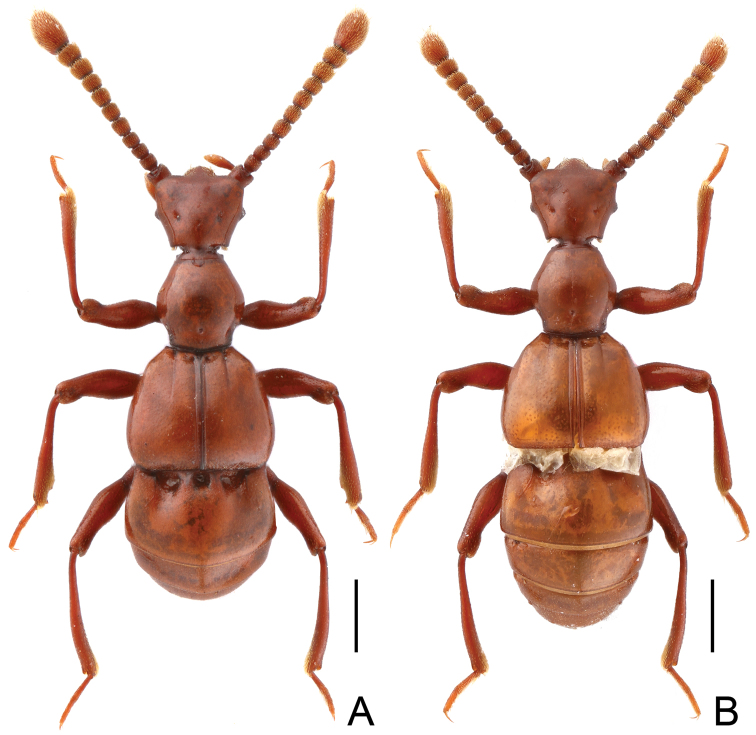
Dorsal habitus of *Tangius indicus*. **A** male **B** female. Scales (mm): 0.5.

**Figure 2.**
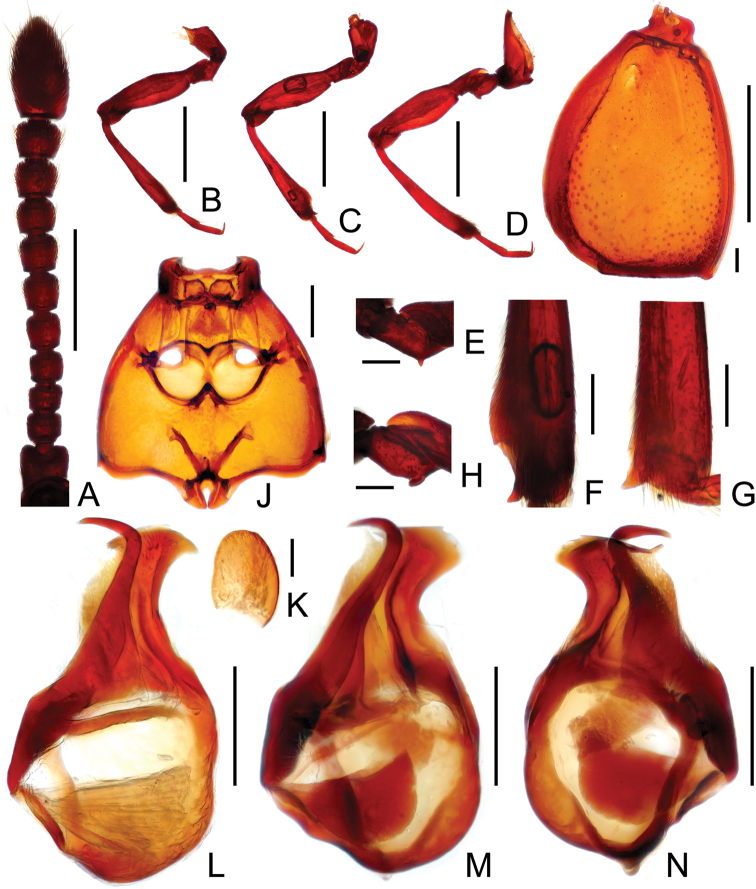
Male diagnostic features of*Tangius indicus* (**A–F, H–K, M–N**) and *Tangius glabellus* (**G, L**). **A** antenna **B** fore leg **C** mid leg **D** hind leg **E** protrochanter **F, G** apical portion of mesotibia **H** metatrochanter **I** left elytron **J** meso- and metaventrites **K** sternite IX **L, M** aedeagus, in dorsal view **N** same, in ventral view. Scales (mm): **A, B, C, D, I** = 0.5; **J, L, M, N** = 0.2; **E, F, G, H, K** = 0.1.

Figs 1 , 2A–F, H–K, M–N

#### Type material

(6 ♂♂, 3 ♀♀). **Holotype: INDIA:** ♂, labeled ‘INDIA, Meghalaya, Khasi Hills, 28.X, Mawphlang, 1800 m, Besuchet-Löbl, 78. / Holotype [red], *Tangius indicus* sp. n., det. Yin & Li 2013, MHNG’. **Paratypes: INDIA:** 4 ♂♂, 3 ♀♀, same label data as holotype [1 ♂ totally disarticulated and preserved in Euparal on plastic boards; 1 ♂ with left antenna and abdomen missing]; 1 ♀, labeled ‘INDIA: Darjeeling, distr., 13 km N. Ghoom, 1500 m, 15.X.1978, I. Löbl, Bes. nr. 15, litter’. Each paratype bear a type label as ‘Paratype [yellow], *Tangius indicus* sp. n., det. Yin & Li, 2013, MHNG’.

#### Description.

Male (Fig. 1A). Length 2.91–2.97 mm. Surface almost glabrous. Head trapezoidal, slightly transverse, HL 0.61–0.62 mm, HW 0.64–0.65 mm; clypeus covered with sparse short setae anteriorly; Antennomeres II–X (Fig. 2A) each subcylindrical, apical antennomeres nearly oval, enlarged. Each eye composed of about 35 facets. Pronotum about as long as wide, PL 0.62–0.63 mm, PW 0.61–0.62 mm; roundly expanded at lateral margins; pronotal apex and base narrowed and truncate. Elytra (Fig. 2I) wider than long, EL 0.79–0.81 mm, EW 1.00–1.03 mm; inner two basal foveae close; discal suture extending to less than half elytral length. Hind wings fully developed. Metaventrite (Fig. 2J) with posterior margin narrowly and deeply notched medially. Protrochanters (Figs 2B, E) with small ventral denticle at middle; mesotibiae (Figs 2C, F) with broadly concave mesal margin before triangular preapical spine; metatrochanters (Figs 2D, H) with blunt ventral projection near apex; all femora with ringed sulcus near base. Abdomen slightly wider than long, AL 0.81–0.83 mm, AW 1.03–1.07 mm; sternite IX (Fig. 2K) nearly oval. Aedeagus (Figs 2M, N) length 0.57 mm; median lobe asymmetric, apex rounded, with sharp, weakly-sclerotized projection at right side; dorsal lobe well-sclerotized, slender, curved rightwards near apex.

Female (Fig. 1B). Identical to male except antennomeres shorter, and mesotibiae lacking distal concavity and preapical tooth.. Measurements: BL 2.73–2.81 mm, HL 0.51–0.52 mm, HW 0.53–0.54 mm, PL 0.53–0.54 mm, PW 0.52–0.54 mm, EL 0.66–0.68 mm, EW 1.00–1.03 mm, AL 0.91–0.95 mm, AW 1.06–1.07 mm.

#### Comparative notes.

The new species is closely allied to *Tangius glabellus* in sharing similar body size, general habitus, including the unusual head shape, and aedeagal structure (Figs 2L, M). Males of these two species can be best separated by the subcylindrical antennomeres VII–X in *Tangius indicus*, and the mesotibiae being broadly concave just before the preapical denticle (Fig. 2G), while *Tangius glabellus* has asymmetric, transversely trapezoidal antennomeres VII–X, and the mesotibiae with straight mesal margins (Fig. 2G). The females of *Tangius indicus* have relatively longer antennomeres than those of female *Tangius glabellus*.

#### Distribution.

North India: Meghalaya, West Bengal.

#### Biology.

One female paratype was collected from sifted litter, as inferred from the label data. Members of this genus are supposed to be inquilines of ants as they exhibit obvious morphological adaptions to myrmecophily, e.g. smooth body surface, compressed antennae, reduction of foveae, etc. The type series of *Tangius glabellus* was collected from the colony of an unidentified ant nesting under a fallen tree.

#### Etymology.

The specific epithet refers to the country where the type series were collected.

## Supplementary Material

XML Treatment for Tangius indicus
